# Effect of Coenzyme Q10 and transcutaneous electrical acupoint stimulation in assisted reproductive technology: a retrospective controlled study

**DOI:** 10.1186/s12958-022-01043-9

**Published:** 2022-12-08

**Authors:** Shanqin Qi, Qi Liang, Lixia Yang, Xueyuan Zhou, Kun Chen, Ji Wen

**Affiliations:** 1 Shandong University of Traditional Chinese Medicine, Jingshi Road, Jinan, 250355 People’s Republic of China; 2 Reproductive Medical Center, the Second Hospital affiliated to Shandong University of Traditional Chinese Medicine, Jingba Road, Jinan, 250001 People’s Republic of China

**Keywords:** *in vitro* fertilization, oocyte quality, optimal embryo, poor ovarian response, pregnancy outcome

## Abstract

**Purpose:**

To investigate the effects of coenzyme Q10 (CoQ10) and transcutaneous electrical acupoint stimulation (TEAS) pretreatment on pregnancy in patients with poor ovarian response (POR).

**Methods:**

A total of 330 POR patients who were pretreated with CoQ10 or CoQ10 combined with TEAS before their in vitro fertilization/intracytoplasmic sperm injection and embryo transfer (IVF/ICSI-ET) cycles and who were not pretreated were selected and divided into CoQ10 group (group A, *n* = 110), CoQ10 + TEAS group (group B, *n* = 110) and control group (group C, *n* = 110). For patients with 2 or more transfer cycles, only the information of the first cycle was included. Ovarian function, response to gonadotropin (Gn) stimulation, and pregnancy outcomes of the three groups were compared in the IVF/ICSI-ET cycles.

**Results:**

After pretreatment, basal FSH, total Gn dosage and duration were comparable among the three groups (all *p*-value > 0.05), basal E2 in group B decreased significantly compared with the control group (*p* = 0.022). Endometrial thickness on the human chorionic gonadotropin (hCG) day, antral follicle counts (AFC), the numbers of oocytes, metaphase II (MII) eggs and excellent embryos in the two pretreatment groups were significantly increased compared with group C (all *p*-value < 0.001), but the rates of MII oocytes, fertilization and excellent embryos had no apparent change. The endometrial thickness on the day of hCG, the numbers of MII eggs and excellent embryos in group B were higher than those in group A (p < 0.001; *p* = 0.020; *p* = 0.027; respectively). The embryo implantation rate (IR), clinical pregnancy rate (CPR) and live birth rate (LBR) in group B were significantly higher than those in group C (*p* = 0.022; *p* = 0.010; *p* = 0.019; respectively), but not significantly different from group A.

**Conclusion:**

CoQ10 alone or in combination with TEAS are effective methods for IVF/ICSI-ET adjuvant therapy, which can significantly improve ovarian reactivity, increase the numbers of retrieved eggs and superior embryos, and improve endometrial receptivity. Adjuvant TEAS on the basis of CoQ10 can significantly enhance pregnancy rates, but CoQ10 alone failed to present such an obvious effect.

## Introduction

10% of women of childbearing age worldwide experience infertility [1]. Various regimens and adjuvant therapies for infertility have been explored and studied. In vitro fertilization/intracytoplasmic sperm injection and embryo transfer (IVF/ICSI-ET) has been a common method and has provided pregnancy options for many infertile patients [2].

Poor ovarian response (POR) refers to poor ovarian reactions to gonadotropin (Gn), an inadequate number of harvested and mature eggs, low fertilization or available embryo rate, and consequently low rates of pregnancy and high proportion of cycle cancellation during IVF/ICSI-ET [3]. About 9–24% of controlled ovarian hyperstimulation (COH) patients are presented as POR [4]. Age is an important independent factor that contributes to POR. As age increases, ovarian reserve and sensitivity to Gn stimulation decrease, meaning the quantity and quality of obtained eggs are reduced. For older infertile women, reproductive clinicians aim to recruit more antral follicles, promote their development and maturation, and thus obtain more high-quality embryos and increase the chances of conception.

In addition to the routine ovulation induction regimens, some adjuvant treatments have attracted extensive attention [5].

Coenzyme Q10 (CoQ10) is a mitochondrial nutrient that can improve mitochondrial function and oocyte quality. It is favored in the reproductive health field because of its convenient oral administration and low price [5, 6]. CoQ10 is the only autosecretory antioxidant and an important component of the mitochondrial respiratory chain. It exists in the inner membrane of mitochondria and transfers electrons and protons in the mitochondrial respiratory chain, maintaining membrane potential stability and promoting adenosine triphosphate (ATP) synthesis. At the same time, it removes oxygen free radicals from tissues and body fluids, prevents protein and lipid peroxidation, and protects mitochondria from oxidative stress-induced damage [7, 8]. The CoQ10 content in human tissues decreases with age, resulting in compromised oocyte quality and development [9, 10]. Researchers have pointed out that CoQ10 supplementation can not only preserve the follicle pool, promote oocyte maturation and ovulation, but also restore mitochondrial gene expression in oocytes and improve mitochondrial activity [6].

Additionally, some auxiliary traditional Chinese medicine (TCM) therapies, such as acupuncture and transcutaneous electrical acupoint stimulation (TEAS), are also used and have previously been shown to improve ovarian reactivity and pregnancy outcomes [11, 12]. The principle of acupuncture is that the stimulation signal is transmitted from peripheral nerves to the central nervous system through stimulation at local acupoints, promoting the release of a variety of bioactive factors and producing physiological effects on the corresponding target organs [13]. Studies have shown that electroacupuncture can increase ovarian blood flow, regulate the HPO axis, menstrual cycle and hormone secretion, and promote follicle development and maturation [13, 14].

In this study, we selected some patients identified as POR who underwent IVF/ICSI-ET in our center, and provided them coenzyme Q10 or coenzyme Q10 combined with TEAS pretreatment, or only conventional COH regimen. We compared the ovarian response and pregnancy outcomes of the three groups, aiming to evaluate the effectiveness of the two adjuvant measures in IVF/ICSI-ET.

## Material and methods

### Patients and study design

This study was approved by the Ethics Committee of the Second Hospital Affiliated to Shandong University of Traditional Chinese Medicine, China (No. SZLL-2022–008-02). Informed consent was obtained from the participating couples. All patient information was anonymous and kept strictly confidential.

Retrospective analysis was made on the data of POR patients who visited the Reproductive Medical Center, the Second Hospital Affiliated to Shandong University of Traditional Chinese Medicine from January 2020 to January 2022 and completed IVF/ICSI-ET during this period. The inclusion criteria were as follows: 1) POR stratified according to the Poseidon classification group 4 [15]: age ≥ 35 years old, with diminished ovarian reserve: AFC < 5, AMH < 1.2 ng/ml; 2) BMI < 30 kg/m^2^; 3) basal follicle-stimulating hormone (FSH) < 18 IU/L; 4) infertility for fallopian tube factor or male factor; 5) experienced a gonadotropin-releasing hormone agonist (GnRH-a) short protocol for COH. The exclusion criteria were as follows: 1) previous surgical history affecting ovarian function; 2) endometrial polyps, submucosal fibroids, endometriosis, and other comorbidities affecting endometrial morphology and function; 3) endocrine, immune, or other systemic complications; 4) chromosomal abnormalities in one or both spouses; 5) receipt of other auxiliary drugs or acupuncture therapy in the past 3 months.

A total of 330 patients were selected. According to the adjuvant treatment schemes, they were divided into CoQ10 group (group A, CoQ10 pretreatment before conventional COH scheme, *n* = 110), CoQ10 + TEAS group (group B, CoQ10 + TEAS pretreatment before conventional COH scheme, *n* = 110) and control group (group C, only conventional COH scheme, *n* = 110).

### Pretreatment schemes

#### CoQ10

CoQ10 (Nengqilang, 10 mg, Weicai Pharmaceutical Co., Ltd, China) was administered orally at a dose of 10 mg, 3 times daily for 2 months before the initiation of the ovulation induction cycle and then for 14 days after transplantation, for a total duration of 3 months.

#### TEAS

Han's acupoint nerve stimulator (HANS, Nanjing Jisheng Medical Technology Co., Ltd) was used for TEAS. Tianshu (ST25), Zigong (EX-CA1), Guanyuan (RN4), Zhongji (RN3), Zusanli (ST36) and Sanyinjiao (SP6) were selected according to TCM theory. TEAS was administered after menstruation at a frequency of 2 Hz and intensity of 15–25 mA, guided by patients tolerance, for 30 min every other day for two menstrual cycles before COH, and until the day of human chorionic gonadotropin (hCG) injection.

### COH and IVF/ICSI-ET protocols

Patients in all groups were given GnRHa short protocol: Triptorelin (GnRHa, 0.1 mg, Ferring Pharmaceuticals, Kiel, Germany) 0.1 mg/d from menstruation cycle day (MC) 2 until hCG administration day. Recombinant FSH (Gonal-F, 150 IU, Merck Serono SA Aubonne Branch) was administered at 150–225 IU starting on MC 3. The initial dose was based on patients’ age, AFC, and BMI. Dosage was adjusted according to follicle growth and serum E2 hormone level after 4 days, and every 1–2 days thereafter. When 2 follicles reached 18 mm in diameter, or 3 or more follicles reached 17 mm, 5000-10000 IU hCG (2000 IU, Lizhu Pharmaceutical Trading, China) was administered to induce ovulation, and oocytes were retrieved 35–36 h later under the guidance of transvaginal ultrasound. Routine IVF or ICSI fertilization was performed according to semen quality. Fertilization and embryo development were evaluated and recorded. On the third day after oocyte retrieval, 1–2 high-quality embryos (grade I/II 8-cell blastomere embryos) were transferred or frozen, and the remaining embryos were placed in extended culture to the blastocyst stage. Only those with good morphology were frozen for subsequent frozen-thawed embryo transfer (FET). A maximum of two high-quality embryos (grade I/II 8-cell embryos on Day 3 (D3) after oocyte retrieval or good-morphology blastocysts on Day 5 or 6) were transferred in a fresh or frozen-thawed cycle after communication, depending on the patients' age, embryo grade, endometrial score and other factors.

Endometrium preparation and FET: Hormone replacement treatment was used in all FET cases. Briefly, oral estradiol valerate tablets (Progynova, 1 mg, Bayer, Germany) 6 mg/d were given starting on MC 3, and the endometrium was monitored by ultrasound starting on the 10th day of the menstrual cycle. When the endometrial thickness reached 8 mm, intramuscular progesterone (20 mg, Zhejiang Xianju Pharmaceutical Co., Ltd, China) 60 mg/d and oral dydrogesterone (Duphaston, 10 mg, Abbott Biologicals B.V., Netherlands) 20 mg/d were administered. D3 embryos were transferred on the 4th day of endometrial transformation and blastocysts were transferred on the 6th day.

From the day of egg retrieval in the fresh cycle or the day of endometrial transformation in the frozen-thawed cycle, 60 mg of progesterone injection and 20 mg of dydrogesterone were administered daily until the day of serum β-hCG test. If pregnancy is confirmed, luteal phase support was continued until 10 weeks of gestation.

### Endpoint measures and definition

Basal FSH and E2 levels, AFC were assessed. Gn dosage and duration, number of retrieved eggs, metaphase II (MII) eggs, fertilized oocytes, and excellent embryos were recorded after oocyte pick-up. Embryo implantation rate (IR), clinical pregnancy rate (CPR), live birth rate (LBR) and early abortion rate were calculated.

The primary endpoints were the number of optimal embryos and CPR, and the secondary endpoints included Gn dosage and duration, numbers of retrieved oocytes and MII oocytes, endometrial thickness, MII oocyte rate, fertilization rate, optimal embryo rate, IR, LBR and abortion rate.

MII oocyte rate = number of MII oocytes / number of retrieved oocytes × 100%; Fertilization rate = number of 2PN oocytes / number of retrieved oocytes × 100%; Optimal embryo rate = number of high-quality embryos / number of D3 embryos × 100%; IR = number of visible gestational sacs on ultrasound / number of transferred embryos × 100%; CPR = number of clinical pregnancy cycles / number of transfer cycles × 100%; LBR = number of delivery cycles of live infants / number of transfer cycles × 100%; early abortion rate = number of cycles of abortion before 12 weeks of gestation / number of clinical pregnancy cycles × 100%.

For patients with 2 or more transfer cycles, only the information of the first cycle is included.

### Statistical analysis

SPSS 27.0 software package (IBM Corp., Armonk, NY, USA) was used for statistical analysis. The numerical variables conforming to the normal distribution were expressed using mean ± standard deviation ($\overline{\mathrm{x} }$ ± s), analysis of variance (ANOVA) was used for comparison between groups, the LSD test was used after ANOVA; Categorical variables were expressed as percentage (%), and comparison between groups was performed using χ^2^ test or continuous correction χ^2^ test as appropriate. A P < 0.05 was considered to convey statistical significance.

## Results

A total of 330 patients were enrolled. The baseline data, ovarian stimulation, pregnancy outcomes and other parameters among the three groups were compared.

### Baseline characteristics

There were no significant differences in age, duration of infertility, BMI and previous IVF failure attempts among the three groups (shown in Table 1).

**Table 1:** Comparison of general data and ovarian stimulation

Characteristics/Variables	Group A (*n* = 110)	Group B (*n *= 110)	Group C (*n* = 110)	*P (ABC)	**P (AB)	**P (AC)	**P (BC)
age (years)	39.22 ± 2.94	39.07 ± 2.91	38.63 ± 2.23	0.244	NS	NS	NS
infertility duration (years)	3.36 ± 2.05	3.55 ± 2.41	3.18 ± 1.74	0.435	NS	NS	NS
BMI (Kg/m2)	22.61 ± 3.05	23.23 ± 3.16	22.77 ± 2.80	0.284	NS	NS	NS
failed IVF attempts (n)	1.39 ± 1.20	1.48 ± 1.19	1.24 ± 1.21	0.309	NS	NS	NS
basal FSH (IU/ml)	12.28 ± 2.78	11.78 ± 2.66	12.43 ± 2.59	0.175	NS	NS	NS
basal E2 (pmol/l)	293.72 ± 74.85	278.51 ± 72.17	301.38 ± 74.58	0.067	0.128	0.442	0.022
AFC (n)	5.69 ± 1.71	6.02 ± 1.64	3.84 ± 1.02	< 0.001	0.104	< 0.001	< 0.001
Gn dosage (IU)	2337.53 ± 482.71	2280.77 ± 460.40	2360.05 ± 637.92	0.525	NS	NS	NS
Gn duration (days)	10.92 ± 2.03	10.68 ± 2.15	11.03 ± 1.77	0.422	NS	NS	NS
endometrial thickness (mm)	9.55 ± 1.18	10.14 ± 1.34	8.95 ± 1.33	< 0.001	< 0.001	< 0.001	< 0.001
retrieved oocytes (n)	5.15 ± 1.61	5.49 ± 1.56	3.41 ± 0.89	< 0.001	0.066	< 0.001	< 0.001
MII oocytes (n)	4.21 ± 1.57	4.66 ± 1.60	2.77 ± 1.09	< 0.001	0.020	< 0.001	< 0.001
optimal embryos (n)	2.00 ± 1.19	2.34 ± 1.31	1.27 ± 0.81	< 0.001	0.027	< 0.001	< 0.001
MII oocyte rate (%)	81.46 ± 16.28	84.74 ± 14.86	80.30 ± 21.36	0.157	NS	NS	NS
fertilization rare (%)	69.96 ± 22.31	73.61 ± 16.30	67.92 ± 25.35	0.145	NS	NS	NS
good-embryo rate(%)	63.94 ± 24.15	66.92 ± 26.88	62.17 ± 29.31	0.434	NS	NS	NS

*AFC* antral follicle count, *BMI* body mass index, *E2* estradiol, *FSH* follicle-stimulating hormone, *Gn* gonadotrophin, *MII* metaphase II

^*^analysis of variance (ANOVA); ** LSD test; *NS* not significant

### Ovarian function, ovarian stimulation and endometrial profile

As shown in Table 1, there were no significant differences in basal FSH and basal E2 among the three groups (*p* = 0.175; *p* = 0.067; respectively), but basal E2 in group B was significantly lower than that in group C (278.51 vs. 301.38, *p* = 0.022). AFC was significantly different among the three groups (*p* < 0.001), group A and group B had markedly more AFC than group C (both *p*-value < 0.001), but there was no significant difference between group A and B (*p* = 0.104).

The total consumption and duration of Gn in the three groups were similar (*p* = 0.525; *p* = 0.422; respectively). On the day of hCG, the endometrium of the two pretreatment groups was significantly thicker than that of the control group (both *p-value* < 0.001), and that of group B is significantly thicker than that of group A (*p* < 0.001).

The number of oocytes retrieved, MII oocytes, and high-quality embryos in group A and B were significantly higher than that in group C (all *p*-value < 0.001). The number of eggs obtained in group A was similar to that in group B (*p* = 0.066), but MII eggs and excellent embryos were significantly different (*p* = 0.02; *p* = 0.027; respectively). There was no apparent change in MII egg rate, fertilization rate and excellent embryo rate among the three groups (*p* = 0.157; *p* = 0.145; *p* = 0.434; respectively).

### Embryo transfer and pregnancy outcomes

Table 2 showed that the three groups completed 102, 104, and 93 ET cycles respectively. The cycle cancellation rate due to the absence of transferable embryos was 7.27%, 5.45%, and 15.45% respectively (*p* = 0.026), with significant difference between group B and group C (*p* = 0.015). There was no significant difference in the numbers of fresh ET cycles and freeze–thaw cycles among the three groups (*p* = 0.661), nor in the types (D3 embryo or blastocyst) or numbers (single or two) of embryos transferred (*p* = 0.763; *p* = 0.108; respectively), and any two groups were comparable.

**Table 2:** Comparison of embryo transfer and pregnancy outcomes

Variables	Group A (*n* = 102)	Group B (*n* = 104)	Group C (n = 93)	^#^P (ABC)	^#^*P* (AB)	^#^*P* (AC)	^#^*P* (BC)
Cycle concellation (n)							
Concel	8	6	17	0.026	0.581	0.056	0.015
Transfer	102	104	93				
Embryo transfer (n)							
Fresh ET	63	68	55	0.661	NS	NS	NS
FET	39	36	38				
D3 embryo	48	44	40	0.763	NS	NS	NS
Blastocyst	54	60	53				
Single embryo	46	43	52	0.108	NS	NS	NS
Two embryos	56	61	41				
Reproductive outcome (%)							
IR	23.40 (37/158)	28.50 (47/165)	17.20 (23/134)	0.071	0.299	0.188	0.022
CPR	30.40 (31/102)	38.50 (40/104)	21.50(20/93)	0.036	0.223	0.158	0.010
LBR	24.50 (25/102)	31.70 (33/104)	17.20(16/93)	0.062	0.249	0.211	0.019
Early abortion rate	16.10 (5/31)	15.00 (6/40)	20.00 (4/20)	0.884	NS	NS	NS

*CPR* clinical pregnancy rate, *ET* embryo transfer, *FET* frozen-thawed embryo transfer, *IR* implantation rate, *LBR* live birth rate

^#^ χ^2^ test; *NS* not significant

The IRs of the three groups were not significantly different (*p* = 0.071), group A was comparable to group B and C, and group B had markedly higher IR than group C (*p* = 0.022). Compared with group C, the CPRs of the two pretreatment groups were significantly increased (*p* = 0.036), in which group B was significantly higher than group C (*p* = 0.010), and the difference between group A and the other two groups was not statistically significant. There was no significant difference in LBR among the three groups (*p* = 0.062), but that of group B was significantly different from group C (31.70% vs. 17.20%, *p* = 0.019), and group A was not significantly different from the other two. The early abortion rates were similar in the three groups (*p* = 0.884).

### Safety evaluation

No local or systemic side effects during treatment were noted in patients receiving CoQ10 and TEAS.

## Discussion

Assisted reproductive technology (ART) breaks the natural cycle pattern where only one dominant follicle develops and matures with ovulation and fertilization. The addition of exogenous Gn promotes more antral follicles to enter the growth and developmental track in the early follicular stage. At the same time, sufficient FSH is also provided to promote follicular development and maturation, which artificially prolongs the FSH window period so that multiple oocytes can mature and fertilize over the course of one COH cycle, which increases the number of embryos and the probability of conception [16]. However, for elderly patients or those with POR, declining ovarian reserve results in a reduced number of antral follicles, and even if a large dose of Gn is given, the number of oocytes can still be insufficient. In addition, high dose FSH accelerates follicular consumption [5]. Therefore, obtaining more mature oocytes and usable embryos is essential to improve the pregnancy rates in POR patients. In addition to routine ovulation induction drugs (i.e. GnRH analogues and Gn), determining the complementary measures to improve ovarian response and pregnancy outcomes is a challenge for reproductive physicians.

In our center, for patients identified as POR, in addition to guiding and adjusting their lifestyle, such as increasing sunlight, exercising moderately, and avoiding staying up late, we will also use some auxiliary treatments, such as growth hormone, CoQ10, TEAS, etc. In this study, we choose CoQ10 and TEAS, retrospectively selected POR patients who entered the COH cycle after pretreatment with CoQ10 or TEAS on this basis, and analyzed the different auxiliary effects on ovarian stimulation and pregnancy outcomes. The results showed that the basal FSH and E2, Gn dosage and induction duration decreased insignificantly after giving CoQ10 for 3 months; AFC increased significantly, showing the trend of improving ovarian reserve. These are consistent with the results of Gat et al. in a large sample size retrospective study. They achieved significantly increased AFC in both IUI and IVF groups and decreased Gn consumption in IVF group after adding CoQ10 on the basis of DHEA, but no obvious change occurred on basal FSH [17]. A Chinese study also achieved significant reduction in Gn dosage after CoQ10 pretreatment [18]. It is speculated that the difference between our results and those above may lie in the different inclusion criteria. They selected POR patients either according to Bologna standard, with lower basal FSH (9.9 vs. 12.43) and higher AFC (5.9 vs. 3.84) than ours; Or Poseidon standard group 3, with younger age (< 35). Unequal ovarian function led to significant difference in responsiveness to Gn. In animal experiments, CoQ10 was reported to reduce the reproductive damage of cisplatin and other toxic drugs in rodents, increase AFC and AMH, and inhibit follicular atresia [19]. It also restored ovarian reserve in a mouse model with rapid follicle loss [6].

Compared with the control group, the significant thickening of endometrium in group A may be related to the increase in the number of mature follicles, the production of more E2, and the promotion of endometrial growth [20].

The numbers of obtained eggs, MII eggs and excellent embryos increased significantly, indicating the improvement of ovarian reactivity and embryogenic capacity. These are similar to the results of Xu et al., they found that the numbers of obtained eggs and excellent embryos, and the fertilization rate increased significantly [18]. In agreement, Gat et al. also calculated significantly increased mature follicles in IUI group [17]. Studies have shown that if exogenous CoQ10 was supplemented in time, damaged oocytes and early embryos could be saved, manifesting as more retrieved eggs and improved embryonic developmental potential, and the decline in fertility could be delayed or even reversed [6]. This is particularly beneficial for older women, as reduced fertility in this group is mainly caused by decreased basal follicles, which reduces the number of retrieved and mature oocytes.

Oocyte growth and maturation, fertilization, and embryo development are highly dependent on adenosine triphosphate (ATP) [21]. As female age increases, oocyte mitochondrial DNA activity and ATP production decreases, resulting in the inhibition of the above-mentioned processes, which weakens the developmental potential of oocytes and embryos [22]. Some researchers suggested that CoQ10 supplementation can improve mitochondrial function, promote ATP production, and increase early cleavage rate and zygote blastocyst formation rate [6, 23].

The more oocytes are developed and the more embryos are formed, the more energy is consumed. If the function of mitochondria is defective and energy generation is insufficient, the ability of oocyte maturation and embryo formation will decline. We obtained MII eggs and excellent embryos in group A much more than those in group C, the rates of MII eggs and excellent embryos remained stable, suggesting that CoQ10 may improve embryo quality by optimizing mitochondrial function and increasing energy output to supply oocyte maturation, fertilization, cleavage and embryogenesis, as well as other important processes.

The IR, CPR, and LBR in group A were higher than those in group C, but not statistically significant. Bentov et al., when studying the effect of CoQ10 on the aneuploidy rate of oocytes, revealed that CoQ10 could increase the pregnancy rate (33% vs. 26.7%) and reduce the aneuploidy rate (46.5% vs. 62.8%), but the difference was not statistically significant. This may be related to the early termination of their experiment due to safety considerations, which only completed 2/3 of the planned sample size [24]. Some studies also pointed out that CoQ10 only increased the numbers of eggs and excellent embryos, but not the pregnancy rate [18]. Whether the reason for insignificant difference on pregnancy results is due to the limited effect of CoQ10 or small sample size or other reasons, which needs to be confirmed in further research.

Traditional Chinese medicine is widely used in our center with satisfactory results, especially for the elderly, POR or patients with previous implantation failure. Acupuncture is a part of TCM therapies, and many studies have confirmed its positive effect on reproduction [11, 12]. TEAS is a modified acupoint stimulation method based on traditional acupuncture. It uses self-adhesive skin electrodes to act on acupoints through precise pulsed electrical stimulation and has the advantages of simple and convenient operation, quantifiable and repeatable treatment parameters, and good patient experience [25, 26]. The effects of TEAS in infertile women, which include improved ovarian response and increased pregnancy rates, have contributed to its popularity in the reproductive field [11, 12, 17–19]. We combined CoQ10 and TEAS in the current study, aiming to complement the advantages of the two adjuvant therapies to improve patients’ fertility.

After adding TEAS on the basis of CoQ10, group B showed a significant reduction in basal E2 and a significant increase in AFC compared with group C, while basal FSH, Gn consumption and duration were comparable among the three groups, indicating that TEAS may improve ovarian reserve and responsiveness to some extent. However, Qu et al. obtained the results that basal FSH and E2, total Gn dosage and time in TEAS groups with different frequencies were equivalent [27]; Shuai et al. also observed insignificant change in basal FSH and Gn dosage and duration in TEAS group [12]. But Zheng’s team revealed that after three courses of TEAS pretreatment, AFC and AMH increased significantly, and basal FSH, E2, FSH/LH decreased significantly. Meanwhile, Gn dosage and time were significantly lower than those of other groups [28]. Differences between studies may originate from different TEAS schemes, including duration and acupoint selection.

Obtained eggs in group B increased significantly, MII eggs and high-quality embryos were markedly more than those of group A. The cycle cancellation rates of group A and group B due to the lack of available embryos were significantly lower than that of group C. It is speculated that TEAS can optimize the microenvironment of follicle development, such as by providing necessary hormone, cytokine and energy support, to promote oocyte fertilization, cleavage, and subsequent embryogenesis and other processes. Studies have confirmed that acupuncture can improve ovarian blood flow and hormone secretion, which are directly associated with oocyte retrieval ability and embryonic potential, thus promoting ovulation and the formation of superior embryos [13, 14, 29]. Consistent with this, some researchers reported significant increase in recovered oocytes and transferred embryos compared with the placebo group and the control group [28], or observed a greater impact of TEAS on retrieved eggs [30]. However, some studies failed to exhibit a significant increase in obtained eggs, MII eggs and excellent embryos [12, 27].

Optimal embryo quality and endometrial receptivity are two prerequisites for successful embryo implantation. Studies have shown that TEAS promotes endometrial growth by upregulating HOXA10 expression and endometrial vascularization. It also inhibits sympathetic excitability, stabilizes uterine muscle activity, increases subendometrial blood flow, and consequently improves endometrial receptivity [31]. Ho et al. found that acupuncture reduced uterine artery resistance and increased uterine blood flow in clinical practice [32]. In the current study, TEAS showed an important role in promoting the growth of endometrium. Similarly, significant improvement in endometrial receptivity in patients with RIF was observed [12]; Others also demonstrated the significant effect of TEAS on endometrium [11, 30].

The IR, CPR and LBR in group B were markedly higher than those in group C, but not significant between group B and A. Chiung et al. achieved statistically higher IR, biochemical pregnancy rate (BPR) and CPR after TEAS administration in patients with ET failures [11]; Significantly increased IR, CPR and LBR were also obtained in other TEAS pretreatment studies [12, 26, 27]. We recognized that these achievements were firstly due to the improvement of ovarian function and responsiveness, more mature oocytes and high-quality embryos were produced, which improved the potential of embryo implantation and continuous pregnancy; And secondly the optimization of endometrial receptivity, which provided premise for improving implantation rate and pregnancy rate.

In addition to analyzing the role of CoQ10 in IVF/ICSI-ET of patients with POR, this study also explored the effect of combined pretreatment of CoQ10 and TEAS, providing ideas for improving the success rate of ART. There are some shortcomings in our study: First, the nature of our retrospective study may compromise the power of the results. Second, the sample size is limited, and there is insufficient ability to analyze the difference of LBR. CoQ10 is taken orally at home and is of high compliance, but TEAS treatment requires hospital visits. Due to the limitations of the epidemic policy, time expenditure and other reasons, the number of patients who completed the 2-month TEAS pretreatment and met the inclusion criteria was limited. However, a larger sample is required to verify the difference of LBR. Third, there's no treatment group that looked at TEAS alone,  which could be used to evaluate the effects of CoQ10 and TEAS separately. What’s more, the specific schemes of CoQ10 and TEAS, such as the selection of acupoints, the optimal dosage and duration of the two treatments, need to be further explored in future practice.

## Conclusion

In the process of IVF/ICSI-ET, pretreatment of CoQ10 or CoQ10 combined with TEAS are effective adjuvant treatments. They can improve ovarian response, increase the numbers of retrieved eggs and superior embryos, and optimize the endometrial receptivity. The addition of TEAS to CoQ10 pretreatment resulted in significant enhancement in pregnancy rates, which was not achieved by CoQ10 alone. In future studies, randomized controlled trials with larger sample size are necessary to fully explain the effects of these two auxiliary measures on reproduction.

## Data Availability

The datasets used and/or analysed during the current study are available from the corresponding author on reasonable request.
